# A degradation debt? Large-scale shifts in community composition and loss of biomass in a tropical forest fragment after 40 years of isolation

**DOI:** 10.1371/journal.pone.0183133

**Published:** 2017-08-23

**Authors:** Rakan A. Zahawi, Federico Oviedo-Brenes, Chris J. Peterson

**Affiliations:** 1 Las Cruces Biological Station, Organization for Tropical Studies, San Vito de Coto Brus, Puntarenas, Costa Rica; 2 Department of Plant Biology, University of Georgia, Athens, Georgia, United States of America; Chinese Academy of Forestry, CHINA

## Abstract

Habitat loss and fragmentation are among the biggest threats to tropical biodiversity and associated ecosystem services. We examined forest dynamics in a mid-elevation 365-ha fragment in southern Costa Rica. The fragment was isolated in the mid-1970s and belongs to the Las Cruces Biological Station. A 2.25-ha permanent plot was established in the center of the old-growth forest (>400 m to nearest edge boundary) and all plants >5 cm DBH were censused, mapped, and identified to species in two surveys taken ~5–6 years apart (>3,000 stems/survey). Although the reserve maintains high species richness (>200 spp.), with many rare species represented by only one individual, we document a strong shift in composition with a two-fold increase in the number of soft-wooded pioneer individuals. The dominant late-successional understory tree species, *Chrysochlamys glauca* (Clusiaceae), and most species in the Lauraceae, declined dramatically. Turnover was high: 22.9% of stems in the first survey were lost, and 27.8% of stems in the second survey represented new recruits. Mean tree diameter decreased significantly and there was a 10% decrease in overall biomass. Such alteration has been documented previously but only in smaller fragments or within ~100 m of an edge boundary. Further penetration into this fragment was perhaps driven by a progressive invasion of disturbance-adapted species into the fragment’s core over time; the loss of once-dominant late successional species could be a contributing factor. The pattern found is of particular concern given that such fragments represent a substantial portion of today’s remaining tropical habitat; further studies in similar-sized fragments that have been isolated for similar prolonged periods are called for.

## Introduction

The large spatial scales at which tropical forest dynamics function present major challenges for conducting appropriately scaled research. Nonetheless, our understanding of such processes has been greatly increased in recent decades with the onset of the first large-scale forest inventories at Barro Colorado Island that were pioneered by Stephen Hubbell and colleagues in the early 1980s [1]. Since then some 63 large-scale permanent plots have been established the world over and are overseen by the Smithsonian Center for Tropical Forest Science (http://www.forestgeo.si.edu/), including a few in temperate areas (see for example [2]). These plots have furthered our understanding of forest dynamics and ecological processes in many ways including seedling recruitment [3, 4], habitat associations of trees [5], shifts in growth rates as a result of climate change [6], and changes in liana abundance [7] among many other topics that have even led to the development of new theories on the maintenance of species richness [8]. However, almost all plots are in intact forest that have not been subjected to major anthropogenic alteration, such as fragmentation. Indeed, with the notable exception of the very well-studied Biological Dynamics of Forest Fragments Project (BDFFP) in Manaus, Brazil [9], few studies have examined long-term forest dynamics in fragmented tropical forests, which represent an increasingly greater proportion of remaining habitat the world over [10].

Remnant forest fragments are increasingly seen to play a critical role in extensively deforested landscapes through the provisioning of ecosystem services [11], as habitat refugia for animals [12–15], as a source of propagules for the restoration of adjacent areas [16], and for mitigating atmospheric carbon accumulation [17]. Despite such important provisions, it is well known that forest fragments are threatened with continued degradation—especially smaller ones where encroachment from the edge can rapidly reduce the remaining core area [10]. Results from the BDFFP have shown that within a 100-m edge buffer there are rapid and abrupt changes in a variety of forest descriptors after fragmentation. Among the well-established phenomena, such edge habitats experience: a) high tree mortality, particularly of large trees; b) an increase in early successional soft-wooded species; c) a reduction of aboveground standing biomass; d) increased tree density; and e) enhanced dynamism [18–21]. Nonetheless, while a few edge parameters such as increased wind disturbance can penetrate >400 m into forest fragments [9], severe edge effects, such as large-scale shifts in community composition, are considered restricted to within the 100 m boundary [22–24]. Studies that have evaluated the interior of forest fragments consider a depth of >200 m to result in little notable impact from edges [23, 25], even when projected 100 years out [26].

In this study, we examined forest dynamics within the core area of the old-growth forest reserve of the Las Cruces Biological Station (LCBS) in southern Costa Rica. The forest fragment was effectively isolated approximately 40 years ago [27], and roughly 200 ha of the reserve is considered old-growth forest. The 2.25 ha LCBS permanent plot was established in the center of the old-growth forest in order to assess the interior dynamics of a medium-sized forest fragment and avoid the afore mentioned and well-documented impacts that occur closer to an edge. We compare a number of community- and population-level parameters over two census periods taken approximately 5–6 years apart. Specifically, we address whether 1) species composition is shifting; 2) certain functional groups are more affected by long-term fragmentation than others; 3) stem density is increasing; and 4) above-ground biomass is changing.

## Materials and methods

### Site location

**Fig 1:**
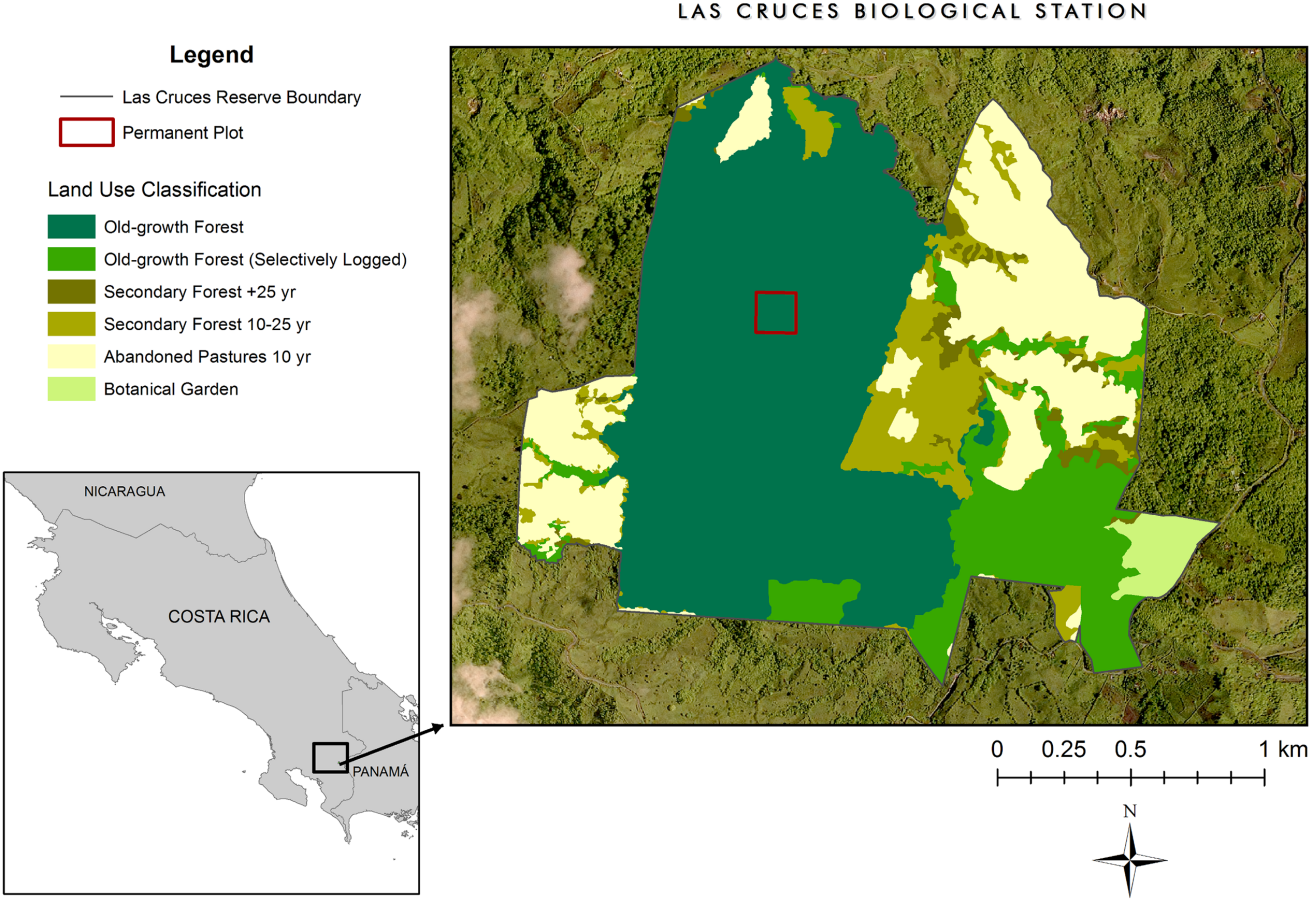
Habitat breakdown of the Las Cruces Biological Station forest reserve in southern Costa Rica and location of the forest dynamics plot. Current and historic edges are clearly visible.

The forest dynamics plot is located at the Las Cruces Biological Station (LCBS; 8° 47' 7" N; 82° 57' 32" W) in southern Costa Rica (Fig 1). The reserve is a remnant fragment that was isolated in the mid-1970s and protects ~200 ha of old-growth forest that has never been logged or burned, as well as a range of other habitats that had been subjected to historical disturbances and are now under various stages of recovery (Fig 1; 365 ha overall). The reserve is classified as a tropical premontane rain forest [28], ranges in elevation from 1000–1430 m asl, and receives a mean annual rainfall of *ca* 3500–4000 mm with a distinct dry season from December to March. Mean annual temperature is ~21°C. The surrounding landscape is a highly fragmented mosaic of mixed-use agricultural fields, pasture, and old-growth and secondary forest patches which account for a little over a quarter of the overall area [27].

### Establishment and census of the forest dynamics plot

Our protocol for surveying the topography and establishing a grid followed that of Condit [29] for the Center for Tropical Forest Science (CTFS) forest dynamics plots. Permission to establish and survey the plot was provided by the Director at LCBS. The center point of the plot (82° 58' 20.769" W; 8° 47' 35.548" N) was randomly placed within the central core area of the Las Cruces old-growth forest (>400 m from a *present-day* edge boundary and >300 m from any historic edge; Fig 1). Thereafter, we established north-south and perpendicular east-west gridlines. From each of a number of reference points, we measured azimuth and distance to the location for each 10 × 10 m grid corner stake (30 cm PVC tubing). Corners were located (x, y, and z coordinates relative to the ‘sighting points’) to within 2–5 cm accuracy using a Topcon CTS-2 surveyor’s total station and reflecting prism (Tokyo, Japan), enabling us to reconstruct the three-dimensional surface of the entire plot (S1 Fig).

The forest dynamics plot was first topographically surveyed in June-July 2007 as a 1-ha plot. The plot was censused between June-September 2008 following which a decision was made to increase the size of the plot to 2.25 ha (150 × 150 m). The remaining 10 × 10 m quadrats were censused between March 2009 and May 2010. Accordingly, we consider the first census to span June 2008-May 2010 (hereafter first census). The plot was recensused between July 2014-May 2015 (hereafter second census).

All woody plants >5 cm in diameter at breast height (DBH; including trees, palms, tree ferns, and lianas) were tagged, inventoried, and their diameter recorded at 1.3 m height; on slopes, the height for diameter measurement was taken on the uphill side. Buttressed trees were measured immediately above buttresses (up to 6 m height). Diameter was measured with a DBH tape or on occasion with calipers. The location of all individuals was mapped to at least 1 m accuracy within each 10 × 10 m quadrat. We followed standard protocols of the CTFS plots (Condit 1998) to sample individuals with multiple and broken stems, to maximize comparability among data sets. Most species were identified in the field or by referencing either the Organization for Tropical Studies Plant Database (www.tropicalstudies.org/plantdatabase) or the Missouri Botanical Garden Database (http://www.tropicos.org/). Species that were not identified to species level were categorized as morphospecies. Permanent aluminum tags, stamped with a unique number, were attached 20 cm above the point of measurement and served as a reference point for future measurement accuracy [30, 31]. In addition, the location of where DBH was taken was marked with red paint.

### Aboveground biomass

Aboveground biomass estimation for trees, palms, and tree ferns was based on the following formula by Chave et al. [32] for tropical moist forests:

$$AGB=\rho \;x\text{ exp}[-1.499+2.148\text{ ln}\left(DBH\right)+0.207{\left(\text{ln}\left(\text{DBH}\right)\right)}^{2}-0.0281{\left(\text{ln}\left(\text{DBH}\right)\right)}^{3}$$

*where* AGB is estimated aboveground biomass in kg; ρ is wood specific gravity (g/cm^3^); and DBH is diameter at breast height. Specific gravity values are compiled from the Global Wood Density database (supplement to [33]), using direct measures for particular taxa when available. When the value for a given taxon was not available, the value for another species in the same genus was used if only one congeneric was listed. If specific gravity values were available for multiple species in the same genus, then an average of these values was used. In the few cases where no congeneric specific gravity was available, the average of other taxa within the same family was used. AGB for lianas was estimated based on the following formula from Schnitzer et al. [34]:

AGB=exp[−1.484+2.657 ln(DBH)]

### Data analysis

As our hypotheses yield expected ‘directions’ (increase or decrease) of change in forest descriptors, we used 1-tailed paired t-tests, to compare metrics between each survey. Paired t-tests were performed at the subplot level (50 × 50 m; n = 9 subplots) in order to account for spatial variation. Based mostly on results from the BDFFP project we expected the following potential shifts in parameters between surveys: density—increase; basal area—decrease; estimated aboveground biomass—decrease; Shannon diversity—decrease; Fisher’s alpha—decrease; species richness—decrease; % pioneers—increase; and mean DBH—decrease. We used a Kolmogorov-Smirnov test to examine if there was a significant change in size (DBH) distributions across all 10-cm classes. Statistical tests were performed with Sigma-Stat 3.5 (Systat, Inc.).

To quantify change in species composition, we calculated similarity between the first and second surveys for each subplot using EstimateS 9.1 [35]. To visualize the direction of compositional change between surveys, we performed a non-metric multi-dimensional scaling ordination (n = 9 × 2) using the number of individuals as the abundance measure, Sørensen as the similarity measure, and 200 random starting configurations. Species with only 1 or 2 individuals were removed prior to ordination, which was done using PC-Ord 5 (MJM Software, Gleneden Beach, OR).

We examined the relationship of mortality to topography (slope and elevation) as follows: using the elevation of each 10 × 10 m cell grid stake, we calculated the slope (degrees) on each of the four edges of a cell and used the steepest slope as an overall measure of the slope of that cell. By choosing the steepest slope our test for a relationship is conservative. Elevation for each cell was calculated as the mean of the elevations of the corner grid stakes. We summed mortality within each cell, and tested for a relationship with slope or elevation as a linear regression.

## Results

**Fig 2:**
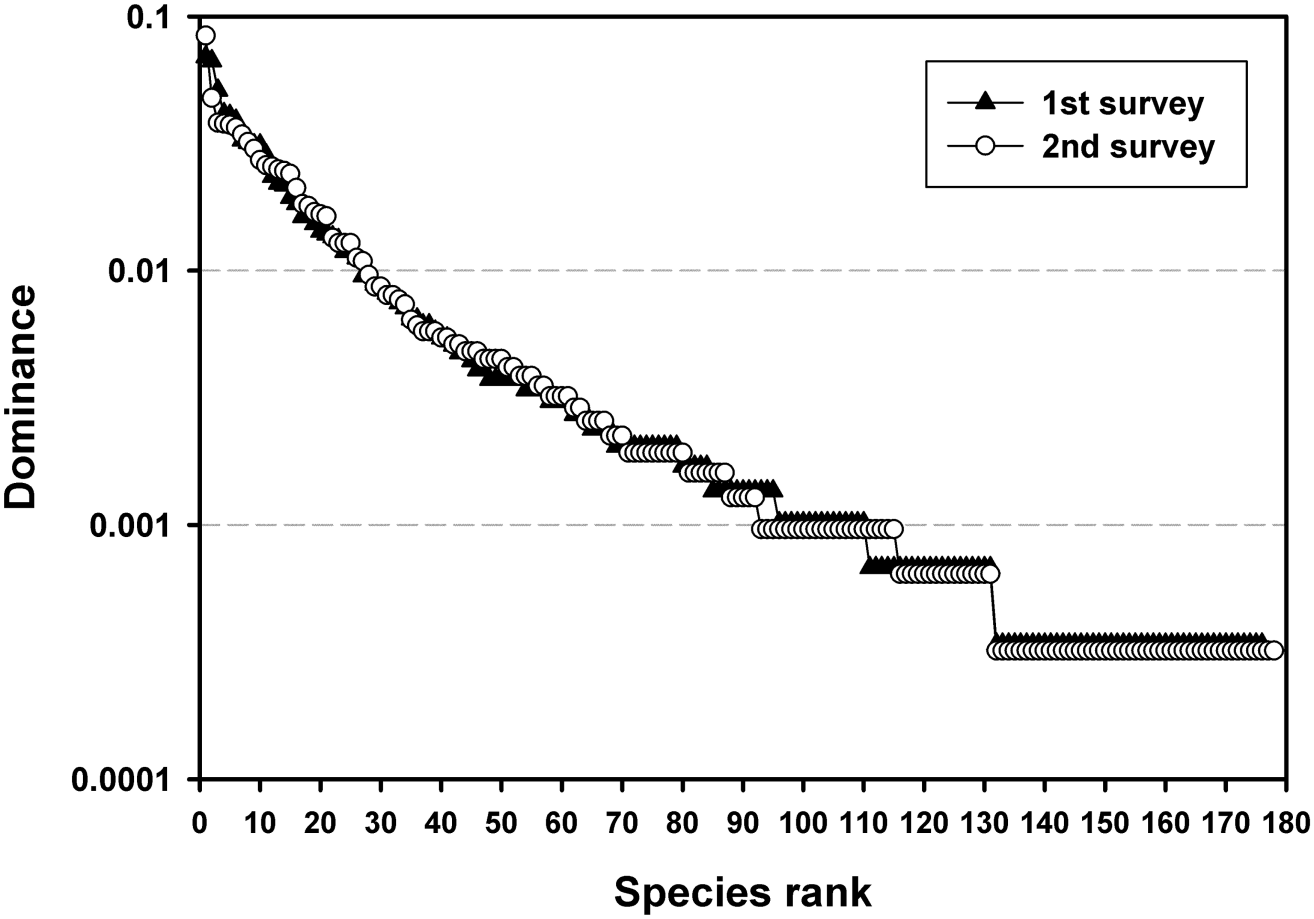
Rank abundances curves for trees censused in each of the two surveys in the Las Cruces forest dynamics plot in southern Costa Rica. Dominance is defined as each species’ proportion relative to the total number of stems ≥5 cm DBH.

More than 3,000 stems were censused in each of the two surveys, representing a little over 200 species including all growth forms (Table 1). The permanent plot captured the majority of tree species registered for the Las Cruces reserve (~260 species censused; [36]), although many species were rare. The vast majority of individuals were identified to species level. Trees that were not identified to at least family/genus level represented 4.8% and 2.4% of each survey, respectively, and were excluded from subsequent analyses. Similar species richness and relative abundance distributions of tree species were reported in both sampling periods (Fig 2). Both surveys had >50 species with only 1 individual censused in the entire plot.

**Table 1:** Summary data collected in the first and second forest dynamics plot surveys in the Las Cruces forest reserve, southern Costa Rica. Total values are for the entire 2.25 ha plot.

	First census (2008–2010)	Second census (2014–2015)
Total number of families/genera (all growth forms)	58/114	59/122
Total number of species (all/trees only)	203/177	206/182
Density—total plot	3113	3316
Density—trees	2835 (91.1%)	3067 (92.5%)
Density—trees (≥30 cm DBH)	240	219
Density—palms	5 (0.2%)	5 (0.2%)
Density—lianas	206 (6.6%)	169 (5.1%)
Density—tree ferns	67 (2.2%)	75 (2.3%)
Basal Area—total plot (m^2^)	80.37	76.20
Basal Area—trees (m^2^)	78.15 (97.2%)	74.46 (97.7%)
Basal Area—trees ≥30 cm DBH (m^2^)	46.21	41.72
Basal Area—palms (m^2^)	0.05	0.04
Basal Area—lianas (m^2^)	1.55 (1.9%)	0.90 (1.2%)
Basal Area—tree ferns (m^2^)	0.61	0.79
Estimated AGB—total plot (Mg)	862.4	777.0
Estimated AGB—trees ≥30 cm DBH (Mg)	644.5	561.1
Mean DBH (cm) (trees only)	14.12 ± 12.25	13.19 ± 11.56
Pioneers (% density/basal area—trees)	3.5%/4.7%	7.2%/5.6%
Number of pioneers (density/basal area—trees)	99/3.68	221/4.19

### Turnover and species composition

Turnover was strikingly high with a total of 713 individuals that were censused in the first survey only (22.9% of all individuals), and a total of 918 new recruits (27.8% of all individuals) in the second survey. Mortality was similar among small and large individuals, with 22.8% of stems <30 cm DBH lost (656 of 2873); and 23.8% of stems ≥30 cm DBH lost (57 of 240).

**Fig 3:**
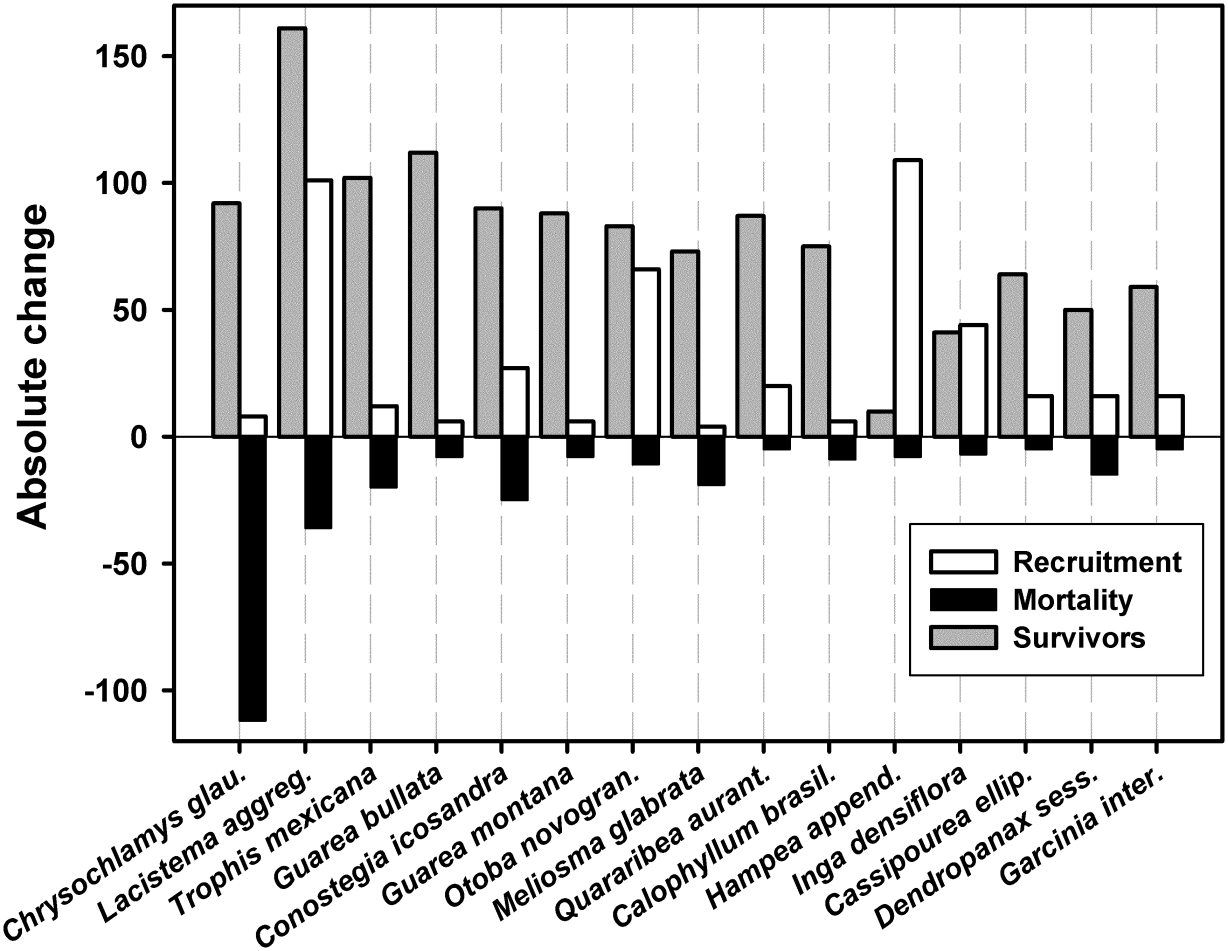
Absolute change in the number of tree recruits and deaths between the two surveys, as well as surviving individuals censused in both surveys, for the fifteen most abundant species (those with a minimum of 60 individuals in at least one of the two surveys).

**Fig 4:**
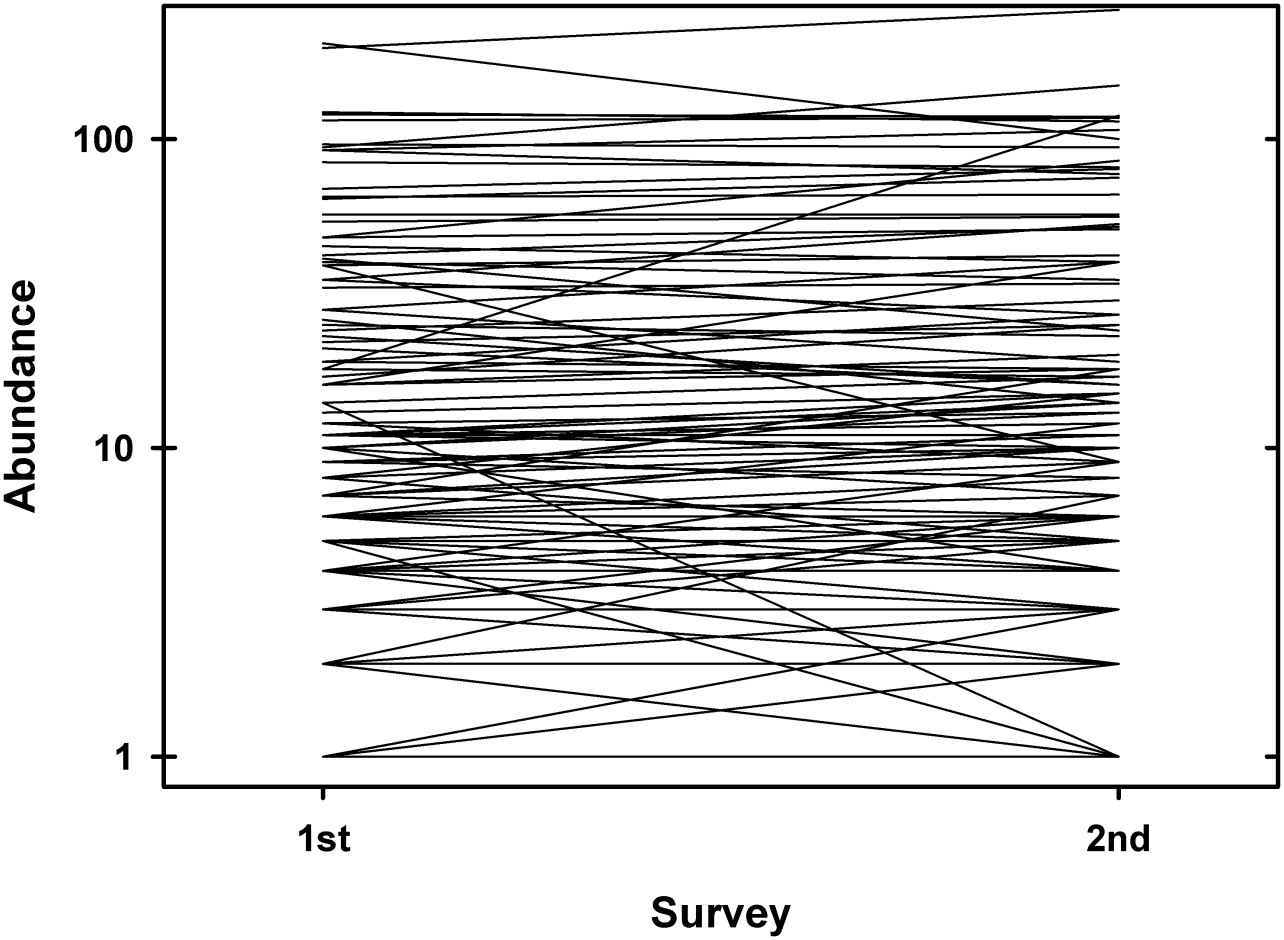
Change in the absolute abundance of all species in the first and second surveys of the Las Cruces forest dynamics plot. Note that if more than one species had the same first and second survey abundances, their lines would be superimposed and appear as one. In addition, as the natural log of zero is undefined, species with starting or ending abundances of zero cannot be shown.

There were notable shifts in species composition between the two surveys. A total of 19 species were censused in the first survey but not the second, and there was a corresponding entry of 22 species in the second survey that were not represented in the first. Bray-Curtis abundance-based similarity varied from 0.762 to 0.857 between the first and second survey in the nine subplots (S1 Table), suggesting that compositional change was of similar magnitude across the entire plot. There were, however, more striking shifts in species composition among dominant species (Fig 3). One late-successional understory tree species, *Chrysochlamys glauca* (Oerst., Planch. & Triana) Hemsl. (Clusiaceae), suffered near catastrophic losses between surveys—it was the most abundant tree in the first survey but lost more than 50% of individuals by the time of the second survey with minimal recruitment of new individuals (Fig 3). Concomitantly, the abundance of a number of early successional species increased dramatically, including *Otoba novogranatensis* Moldenke (Myristicaceae), *Hampea appendiculata* (Donn. Sm.) Standl. (Malvaceae), and to a lesser extent *Lacistema aggregatum* (P.J. Bergius) Rusby (Lacistemataceae). For remaining species, most changes were modest, however, several other intermediate-abundance species had large relative shifts (Fig 4).

**Fig 5:**
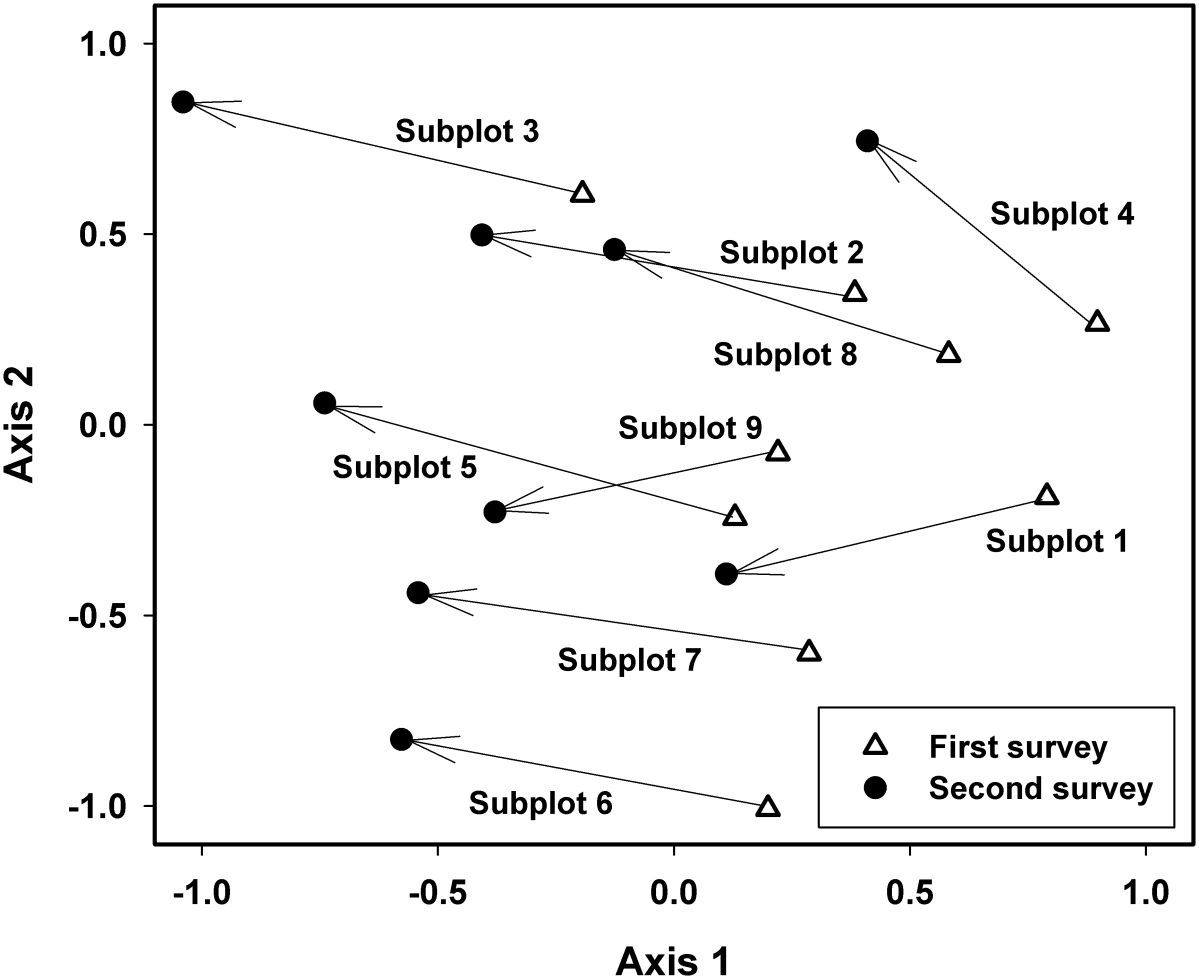
Nonmetric multidimensional scaling assessing the change in species composition at the subplot level (50x50 m) and based on the number of individuals of each species. Species with 1–2 individuals were excluded from analyses.

Change in species composition was visualized via NMDS, and showed several noteworthy patterns (Fig 5). First, all subplots shifted in a similar direction, regardless of starting point. Second, the length of the change vectors was remarkably consistent, indicating (as does S1 Table) that species compositional change was similar and widespread throughout the FDP and not isolated in patches. We also assessed mortality relative to the characteristics of each 10 × 10 m cell. Mean cell elevation varied by >80 m with some located on exposed ridgetops and others along creek beds. The FDP exhibited steep topography and cells varied in slope from 0° to >70° (S1 Fig). However, only a minimal amount of the variance in mortality was explained by elevation (R^2^ = 0.021, *P* = 0.028) and none by slope (R^2^ = 0.0001, *P* = 0.728).

**Fig 6:**
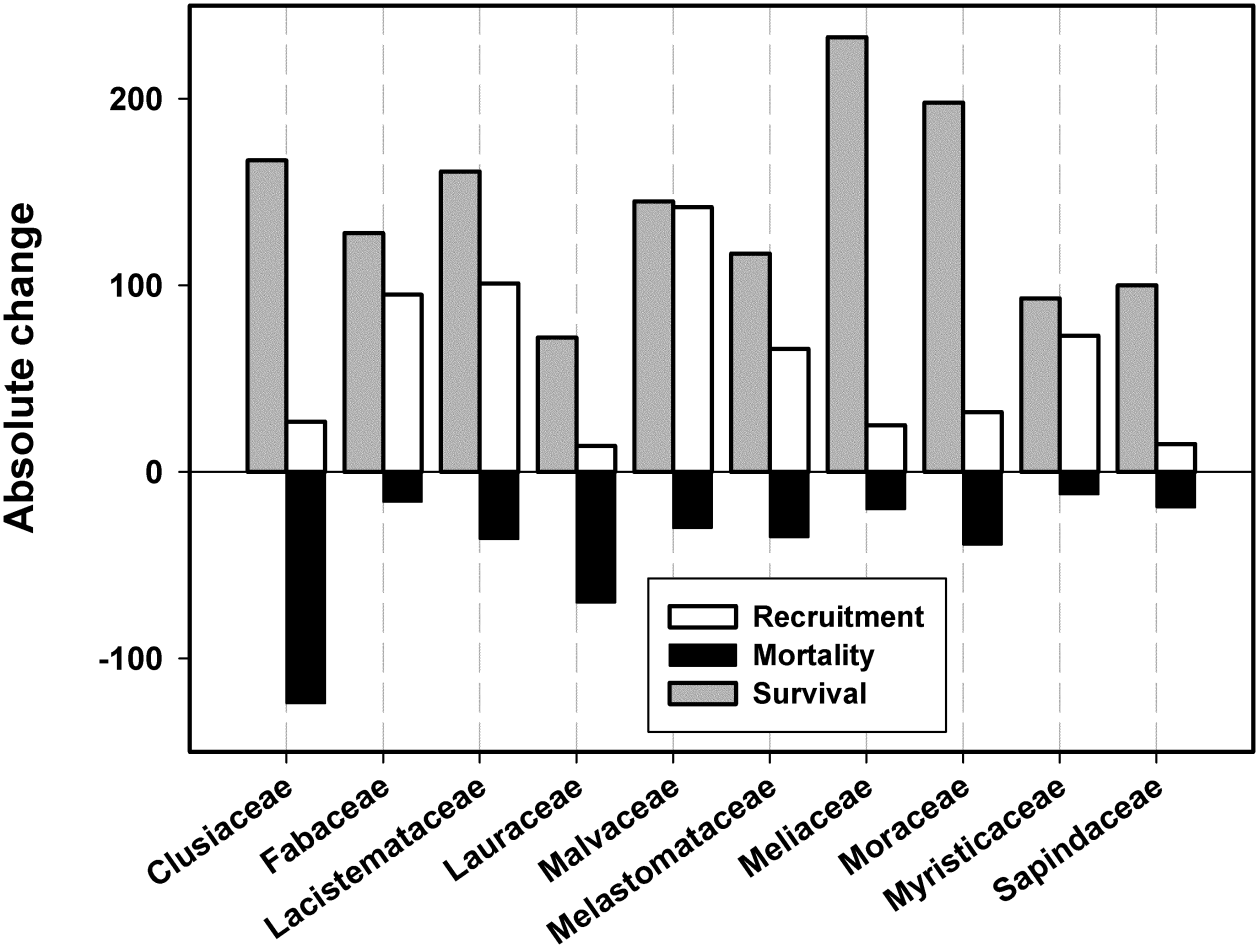
Absolute change in the number of recruits and deaths between surveys, as well as surviving individuals censused in both surveys, for the ten most abundant families (based on the first survey) of the Las Cruces forest dynamics plot.

Strong shifts in composition at the family level were also found (Fig 6). Whereas shifts in a number of families are attributable to changes in the abundance of a single species, such as the decline of *C*. *glauca* and the dramatic increase of *H*. *appendiculata*, two families reflected increases driven by several species within the family (Fabaceae and Melastomataceae), as did the more worrisome decline in the Lauraceae (Fig 6). Approximately half of the 142 Lauraceae individuals censused in the first survey died. Individuals spanned all size classes but a slightly higher proportion of individuals in the mature size classes (those ≥30 cm DBH) died (~57%). The number of Lauraceae species censused also decreased from 26 to 22, and there was comparatively little recruitment (14 individuals) in the second survey. Overall, families that tended to increase in this study are largely represented by early successional species, with the exception of Fabaceae, whereas those that decreased (Clusiaceae and Lauraceae) are represented more by late successional species.

### Density and biomass

There was a 6.6% increase in tree density between the first and second surveys (T = -2.77, df = 8, *P* = 0.012); more than half of the increase was driven by pioneers that increased from 3.5 to 7.2% of total stems (T = -2.93, df = 8, *P* = 0.009; Table 1). Shannon diversity indices increased for all growth forms between surveys (T = -2.56, df = 8, *P* = 0.017), but not for trees (T = 0.10, df = 8, *P* = 0.461), whereas Fisher’s alpha did not change significantly in either case (T <1, *P* >0.5).

**Fig 7:**
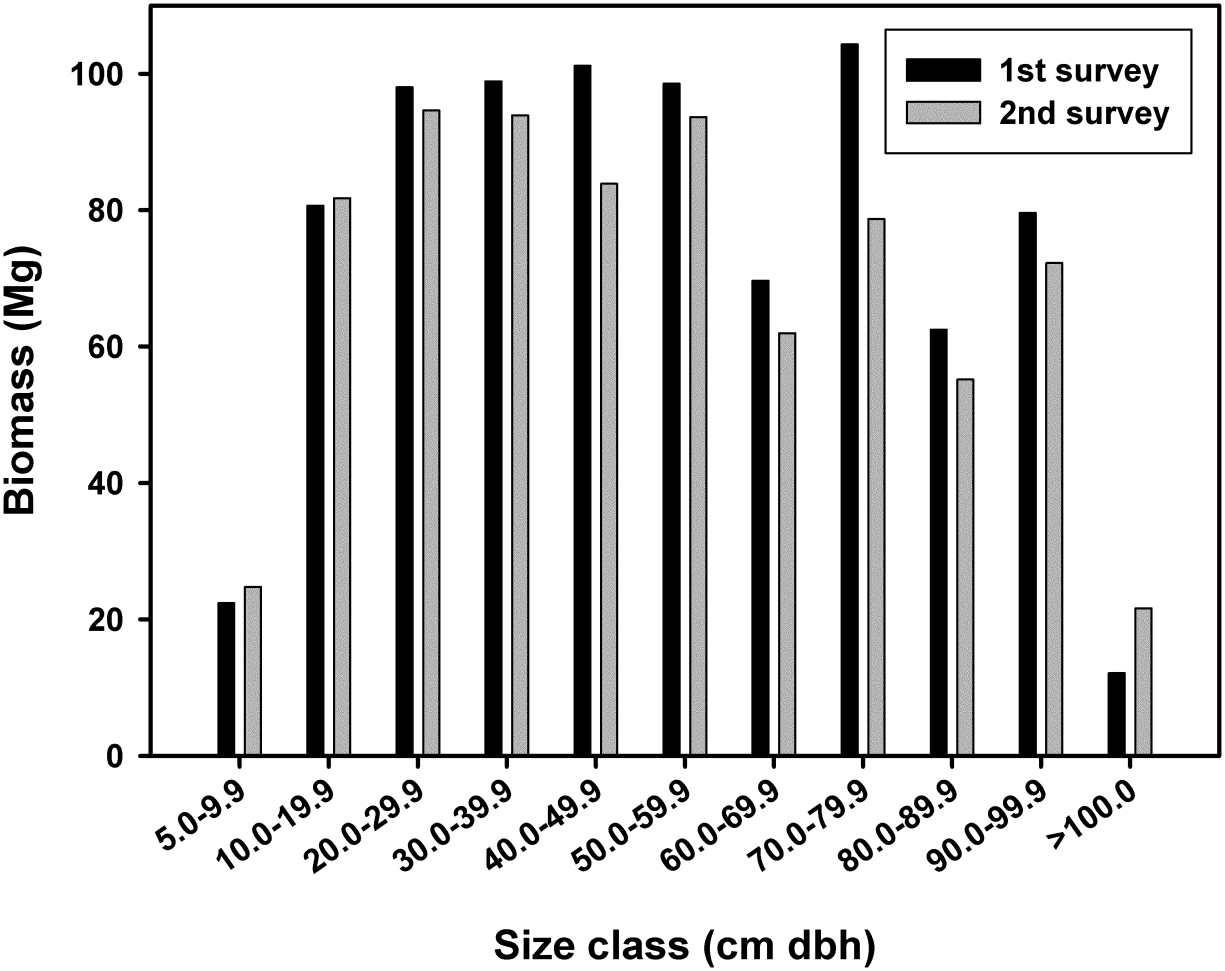
Tree biomass grouped by size class for the first and second surveys of the Las Cruces forest dynamics plot.

Although there was a significant increase in stem density, total above-ground biomass in the plot decreased significantly from 862.4 Mg in the first survey to 777 Mg in the second (T = 2.68, df = 8, *P* = 0.012), representing a ~10% overall decrease. A similar decrease was found if the analysis was repeated for trees only (T = 2.20, df = 8, *P* = 0.029), as most of the lost biomass was held in late-successional trees of the larger size-class categories with denser wood (trees >40 cm, Fig 7). A significant difference in size class distribution was found between the two surveys (K-S = 0.05, *P* = 0.0037), with a higher concentration of stems in smaller size classes in the second survey (S2 Fig), and a corresponding reduction in the mean overall DBH of trees (T = 5.81, df = 8, *P* = 0.0002; Table 1). Interestingly, overall basal area was only marginally lower between surveys (T = 1.72, df = 8, *P* = 0.062 all species; T = 1.56, df = 8, *P* = 0.078 trees only).

## Discussion

The 200-ha old-growth forest core of the Las Cruces reserve harbors a high diversity of tree species with at least 260 reported and more than 340 species possible based on regional censuses [36]. The two surveys of this forest dynamics plot captured a significant proportion of this diversity, although many species were rare and represented by only one individual. The more recent survey, however, found a substantial and consistent shift in species composition across the entire plot for both abundant and rare species and, more worrisomely, a dramatic two-fold increase in the number of soft-wooded pioneer individuals. This shift in composition occurred some 40 years after initial isolation, which suggests that the fragment has not reached a stable state and calls into question whether the fragment can maintain a significant proportion of the characteristic high regional biodiversity over the long-term. Concomitant with the dramatic shift in composition, was a 10% decrease in overall biomass between surveys, and a shift in the size class distribution of trees towards a high concentration of stems in smaller size classes, as would be expected in a forest with high levels of turnover. Loss of biomass after forest fragmentation is well documented across long-term studies of fragmentation [37], and is largely driven by the death of big trees [20], but this has typically been noted within 100 m of a forest edge and within the first two decades after fragmentation [19]. In this study mortality was not concentrated in the largest size classes (>70 cm DBH). Instead, the combination of high tree loss in the 30–70 cm DBH size range, coupled with abundant recruitment of small stems, drove the changes noted between the two surveys.

The old-growth core of the Las Cruces forest fragment was effectively isolated in the mid- to late-1970s, when most forested land in the immediate surrounding area was converted to pasture [27]. Although sections of this original forest perimeter have been buffered more recently by the acquisition of properties that have reverted to secondary forest (primarily the eastern boundary), other aspects of the reserve have essentially maintained the same forest/edge boundary since isolation. In this study, we were careful to select a site where the shortest distance from a clearly defined *present day* edge (pasture matrix) was at least 400 m, and >300 m from a historic edge boundary (only the eastern edge from time of isolation until approximately the year 1998). Whereas a few studies have noted that increased tree mortality can penetrate up to 300 m from a forest edge [20], most have shown that the vast majority of edge-driven deleterious effects do not surpass the 100 m mark(see for example [18, 38]). Accordingly, the Las Cruces forest dynamics plot should have been buffered from the most severe abiotic edge parameters.

When the Las Cruces forest fragment was isolated, there was likely to have been a sharp change in species composition, and an increase in the mortality of larger trees up to 100 m [9, 20, 39]. This dynamic is likely to have gotten progressively more severe as continued alterations in composition within edge habitats many decades after fragmentation have been observed [40]; indeed, reaching a stable state in edge habitat appears elusive. Though further penetration is not well documented, Oliveira et al. [41] observed that fragments up to 300 ha had lost most of their large trees in an expansive study on a suite of fragments isolated more than a century ago in the Mata Atlântica of Brazil. Causality was not clear, however, and logging is considered to have been the likely main driver behind the loss of large trees in the core area of these fragments. In our study, logging was discarded as a possibility as the fragment is well protected as a private reserve.

Several studies have noted striking shifts in selection pressures on flora over short time scales at both the population- [42] and community-level [43] due to the loss of key dispersers, and/or shifts in abundances of seed predators [44]. This is especially of concern in fragmented landscapes. Whereas large frugivores including megafauna such as tapirs and two of four species of monkeys have been locally extirpated in our fragment [45], many other large- to medium-sized frugivores such as toucans (*Ramphastos*), chacalacas (*Ortalis*), and guans (*Penelope*), among others as well as bats persist and in many cases are considered abundant [14, 15, 46, 47]. In turn, a number of surveys indicate that the population densities of seed predators at Las Cruces (such as rodents) are low, likely due to the persistence of cat populations and other faunal predators [45]. Accordingly, although we cannot entirely discount that shifts in the population sizes of seed dispersers and predators could have exacted some changes in forest community dynamics at Las Cruces after isolation, we do not believe this to be the primary driver of results observed. This leaves a combination of several other potential mechanisms; three are outlined here.

First, the deleterious effects of forest fragmentation may have surpassed the 100-m edge boundary given the later-stage time frame of this study. Once pioneer trees, which tend to proliferate after fragmentation within 100 m of an edge [21] reach reproductive maturity, they may be more able to take advantage of an interior gap because of greater proximity to the fragment core; indeed, a few adult pioneers were censused in the FDP lending support to this idea (Zahawi pers. obs.). This pattern has been documented in small fragments of the BDFFP where juveniles of disturbance-recruiting palm species showed a marked increase in abundance 10–15 years after fragmentation [48]; similar results have been reported in fragments (up to ~80 ha) in the Mata Atlântica of Brazil that were isolated >60 years ago [25, 49]. The implications are that disturbance-adapted species account for an increasing proportion of species composition over time, and in this case 35–40 years is sufficient to get disturbance associated successional species (that can reach reproductive maturity within a decade) into the center of a forest fragment—but this has occurred in a substantially larger fragment than has been reported previously. The effect may be further exacerbated in landscapes with an abundance of secondary forest patches dominated largely by pioneer species [50], which is the case here [27]. Indeed the eastern portion of the reserve that has been recovering since 1998 is now dominated by reproductive early-successional species [51].

Alternately, abiotic edge effects could have penetrated the core of this fragment from the onset of regional fragmentation, impacting the recruitment abilities of later successional species and their ability to establish or compete with heterospecifics. Depth of edge is highly variable depending on the parameter measured [9] and the impact of edges is more pronounced if there is more than one edge aspect to facilitate penetration into the core [40], as is the case with this isolated reserve. This penetration may be even more pronounced in our area given the extremely rugged landscape which could allow for increased penetration of variables such as temperature and wind by effectively ‘skipping’ over the buffer forest habitat that surrounds the core area of this fragment. A significant portion of this FDP was located on a ridge-top (S1 Fig) which would lend support to this idea. Additionally, edges in this study are very pronounced (or harsh) which is more detrimental from an abiotic perspective as a pastoral matrix does not provide any buffering [10, 40]. However, our analyses of slope- and elevation-based mortality revealed no relationship in this study, suggesting that if topography influences mortality, such effects are subtle and are likely not linked to abiotic edge penetration.

Finally, climate change could be driving observed changes in forest dynamics and community composition, which may then favor the establishment of disturbance-adapted species. For example, several studies have shown that larger trees are more susceptible to increased drought [52–54] and anomalies in rainfall patterns have increased mortality rates in Amazonian forests [55]. In this study, the widespread mortality of once-dominant species, such as *C*. *glauca*, could create a mechanism for the incursion of disturbance-adapted species. Although the actual cause behind the sweeping mortality of *C*. *glauca* is not known, it is likely due to pathogens (Zahawi pers. obs.). Whereas most pathogen studies are focused at the seed and seedling demographic stage [56, 57] others have documented adult tree susceptibility [58]. Furthermore, the impact of climate change on pathogen behavior and the susceptibility of tropical trees is unclear but it is anticipated to increase [59–61]. If this were the case, we would anticipate similar species composition shifts in nearby intact forest, but this has not been investigated. Nonetheless, a number of studies have noted higher than predicted shifts in species composition in intact forest in other tropical regions and have attributed this to climate change [62, 63]. Similarly, climate-change driven mortality events may be driving the substantial turnover in our forest, which may in turn favor invasion by disturbance-adapted species.

Aside from *C*. *glauca*, there were dramatic shifts in species composition at the family level between the two censuses. The apparent decrease in abundance of Lauraceae individuals should be of particular concern, given that this is a pre-montane wet forest and the dependence of fauna on this family is well known [64]. Decline of wild populations of Lauraceae has been noted recently in temperate regions of the south-eastern US [65, 66], but there have been no noted impacts on populations in tropical regions. Loss of Lauraceae individuals in this study spanned the spectrum of tree size classes with minimal recruitment and, given the pattern of loss documented, it is likely that additional species will be lost in upcoming years. Liana density also decreased between surveys which may seem surprising given that densities of lianas have been reported to increase in both fragments and intact tropical forests [67, 68]. However, the death of a number of large lianas in this study was clearly linked to falling big trees (Zahawi pers. obs.), and this is reflected in the greater proportional loss in liana basal area between surveys. Lastly, whereas shifts in once-abundant species, such as *C*. *glauca*, or changes at the family level are easy to discern, of more concern is the extreme difficulty in quantifying change for rare species. More than 50 species were represented by only 1 individual in this study and a number of these species were censused in only one of the surveys, making it impossible to ascertain whether the population dynamics of such species are stable or not, though their long-term survival in such fragments is likely disfavored [39]. Permanent plots are in all probability not the correct approach to assess the health of such species, and undertaking targeted field surveys of rare individuals is a more robust means of evaluating the persistence of their populations over the long-term.

## Conclusion

Although it is not possible to separate edge-driven impacts from climate change driven parameters, and indeed the changes quantified here may be driven by a combination of several factors, there is no doubt that fragment degradation increased in the time interval between the two censuses detailed here, and is widespread throughout the plot irrespective of slope or elevation. This lag in adjustment, or a species composition degradation debt some 40 years after fragment isolation, is akin to the extinction debts anticipated and documented at the landscape scale in severely fragmented regions of the Mata Atlântica of Brazil [69, 70], where reaching a so-called equilibrium takes on the order of several decades or more. Indeed Haddad et al. [37] in their synthesis paper examining long-term fragmentation studies noted that an extinction debt occurs more rapidly in small fragments and that there is a delayed response within larger forest fragments of similar size to the Las Cruces reserve, but this was largely determined for fauna. Laurance et al. [40] noted, however, that even 30+ years on there were still changes going on in fragments in the BDFF and that a stable state had not been reached, although once again shifts in tree community composition were largely documented within the 100 m edge habitat [18].

Results from this study shed a disconcerting light on the stability of at least this medium-sized tropical forest fragment over the long-term, and on its ability to maintain the high level of diversity typical of these biomes. It calls into question the degree to which a core area can be ‘buffered’ from edge effects or climate change, at least in a rugged landscape such as this one that is dominated by secondary forest patches [27]. This result is particularly noteworthy given that fragments of this size make up an increasingly significant portion of remaining forest habitats in tropical regions [10, 37], and additional studies to evaluate forest dynamics in similar-sized forest fragments in the tropics that have been isolated for prolonged periods are called for.

## Supporting information

S1 Table Similarity indices showing mean values for all subplot comparisons within each of the two surveys (n = 36 each survey), and values comparing similarity between the two surveys for a subplot of the forest dynamics plot at the Las Cruces forest reserve, southern Costa Rica. (DOCX)

S1 Fig Three-dimensional layout of the Las Cruces forest dynamics plot showing slope and relative elevation change. (TIF)

S2 Fig Size class distributions for the number of tree stems censused in the first and second surveys of the Las Cruces forest dynamics plot. (TIF)
