# Supplementary material for: A degradation debt? Large-scale shifts in community composition and loss of biomass in a tropical forest fragment after 40 years of isolation
Source: PLoS One. 2017 Aug 23;12(8):e0183133. doi: 10.1371/journal.pone.0183133 (PMC5568379; doi:10.1371/journal.pone.0183133)
Supplement: S1 Table — (DOCX) [file pone.0183133.s001.docx]

**Table S1.** Similarity indices showing mean values for all subplot comparisons within each of the two surveys (n = 36 each survey), and values comparing similarity between the two surveys for a subplot of the forest dynamics plot at the Las Cruces forest reserve, southern Costa Rica.

| Comparison | Jaccard | | Sørensen | Chao Jaccard raw | Chao Sørensen raw | Bray-Curtis |
| --- | --- | --- | --- | --- | --- | --- |
| Mean (± 1SD) 1^st^ Survey | | .453 ± .031 | .623 ± .029 | .732 ± .029 | .845 ± .019 | .596 ± .049 |
| Mean (± 1SD) 2^nd^ Survey | | .463 ± .039 | .632 ± .036 | .743 ± .043 | .852 ± .028 | .586 ± .052 |
| Subplot 1 | | .793 | .885 | .934 | .966 | .830 |
| Subplot 2 | | .787 | .881 | .922 | .959 | .795 |
| Subplot 3 | | .696 | .821 | .903 | .949 | .782 |
| Subplot 4 | | .752 | .858 | .904 | .949 | .814 |
| Subplot 5 | | .761 | .864 | .919 | .958 | .814 |
| Subplot 6 | | .769 | .870 | .924 | .960 | .830 |
| Subplot 7 | | .792 | .884 | .920 | .958 | .822 |
| Subplot 8 | | .723 | .839 | .913 | .954 | .762 |
| Subplot 9 | | .825 | .904 | .942 | .970 | .857 |
| Mean (± 1SD) 1^st^/2^nd^ Survey | | .766 ± .039 | .867 ± .025 | .920 ± .013 | .958 ± .007 | .812 ± .028 |
