# Supplementary figures and images for: A degradation debt? Large-scale shifts in community composition and loss of biomass in a tropical forest fragment after 40 years of isolation
Source: PLoS One. 2017 Aug 23;12(8):e0183133. doi: 10.1371/journal.pone.0183133 (PMC5568379; doi:10.1371/journal.pone.0183133)

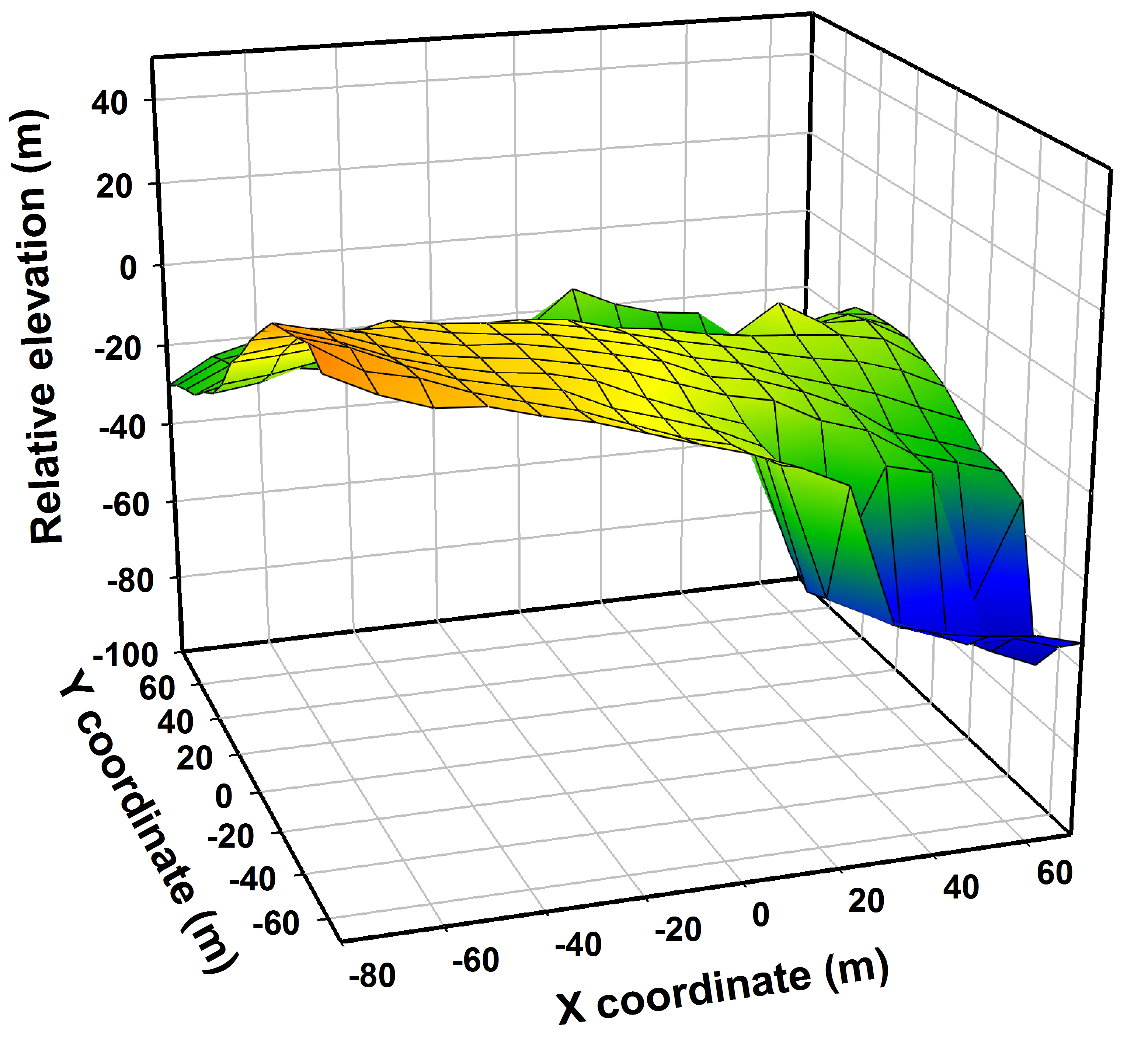

Supplement: S1 Fig — (TIF) [file pone.0183133.s002.TIF]

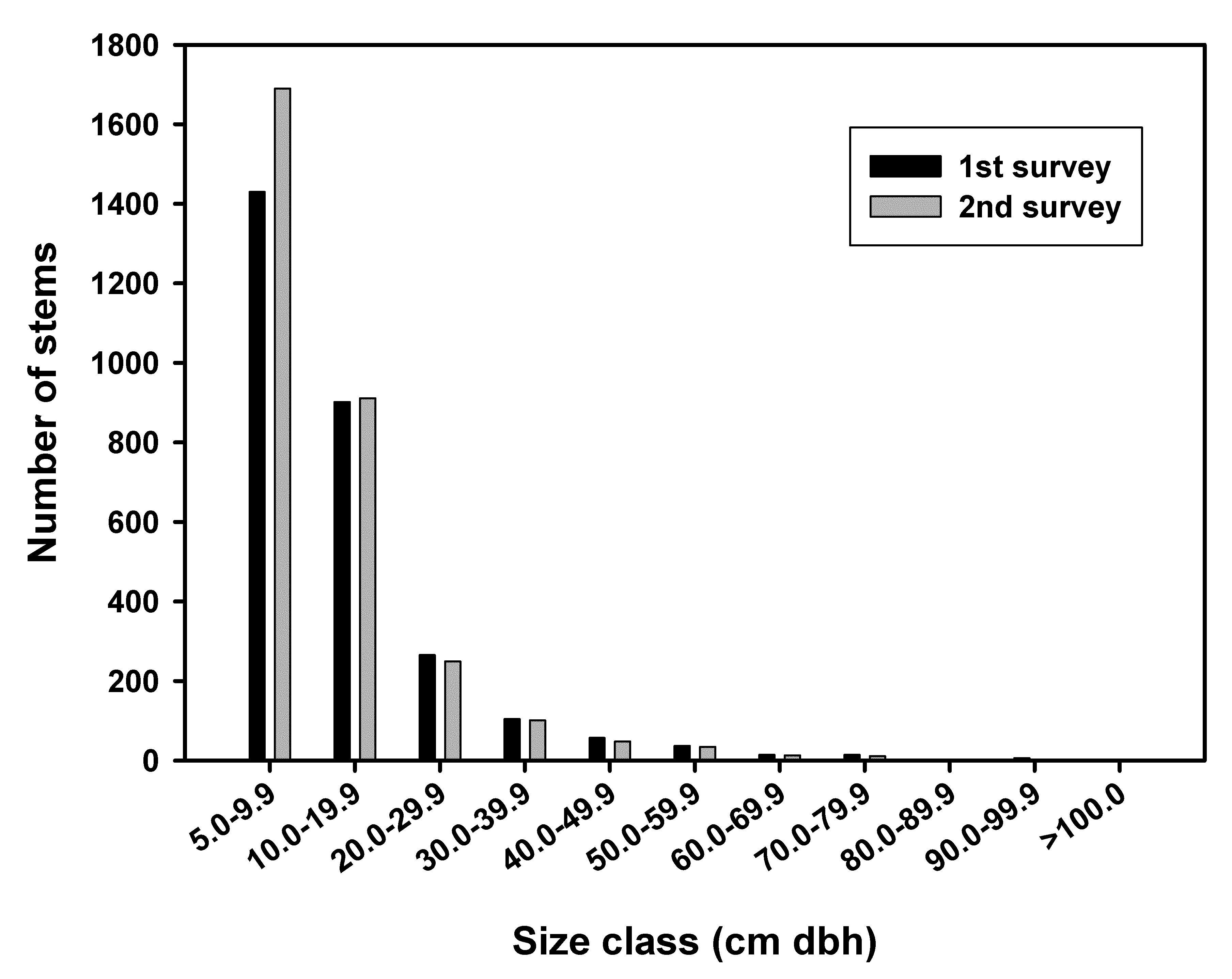

Supplement: S2 Fig — (TIF) [file pone.0183133.s003.TIF]
